# Self-Inflicted Laser-Induced Retinopathy

**DOI:** 10.3390/diagnostics14040361

**Published:** 2024-02-07

**Authors:** Ninel Z. Gregori, Louis Cai, Yasman Moshiri

**Affiliations:** 1 Department of Ophthalmology, Miami Veterans Affairs Medical Center, Miami, FL 33125, USA; louiscai@med.miami.edu (L.C.); yxm685@med.miami.edu (Y.M.); 2 Department of Ophthalmology, Bascom Palmer Eye Institute, University of Miami Miller School of Medicine, Miami, FL 33136, USA

**Keywords:** full-thickness macular hole, macular hole, laser retinopathy, optical coherence tomography, OCT

## Abstract

This photo essay details a patient with self-inflicted laser-induced retinal injury progressing to full-thickness macular holes in both eyes. A 40-year-old patient presented after a self-inflicted injury by a handheld class 3 blue laser (450 nm) he purchased on the internet. The patient reported shining the laser through a window, which reflected the beam back into his eyes. Visual acuity was measured at 20/400 in both eyes. The initial fundus photographs revealed vitreous and preretinal hemorrhages in the right eye, and multiple yellow-white fresh laser burns in the macula of the left eye. Optical coherence tomography (OCT) showed preretinal hemorrhage in the right eye and retinal disruption with preretinal hyper-reflective lesion in the left eye. After one month, his vision deteriorated to finger counting in each eye. He developed a full-thickness macular hole and hyperfluorescent curvilinear streaks in the superior maculae in both eyes. OCT images showed retinal pigment epithelium clumping and outer retinal atrophy in curvilinear streak areas in both eyes, which point to self-inflicted injury. This case illustrates laser-pointer-induced retinopathy and reinforces the necessity of public education on the dangers of utilizing handheld lasers without eye protection.

**Figure 1:**
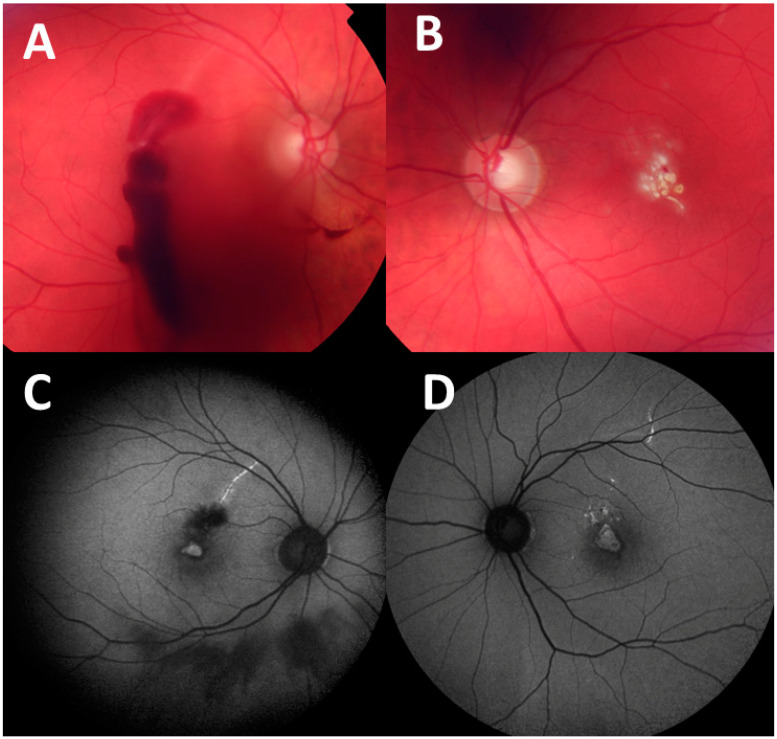
A 40-year-old patient presented after a self-inflicted injury by a handheld class 3 blue laser (450 nm) he purchased on the internet. The patient reported shining the laser through a window, which reflected the beam back into his eyes. The patient’s initial vision was 20/400 in both eyes. Fundus photographs taken at presentation show: (**A**) vitreous and preretinal hemorrhages in the right eye and (**B**) multiple yellow-white fresh laser burns in the central macula of the left eye. One month later, the patient was only able to count fingers with each eye. Fundus autofluorescence images taken at 1-month visit demonstrate hyperfluorescent curvilinear streaks in superior maculae in the right eye (**C**) and left eye (**D**). as well as hyperfluorescence in the fovea of both eyes. Retinal streaks point to self-inflicted laser injury, while peer-inflicted laser injuries have been described to result in focal foveal lesions [1]. Retinal damage from laser pointers may vary in morphology and include subretinal or intraretinal hemorrhage, retinal edema, retinal pigment epithelium scarring, foveal granularity, vitreous or chorioretinal hemorrhage, perifoveal drusenoid-like deposits/pigment clumps, ring-shaped hypopigmented lesions in fovea, macular holes, and rarely, choroidal neovascularization [2]. The Food and Drug Administration (FDA) have identified 4 major classes of lasers (1–4), of which laser pointers belong to class 3R [3]. Exposures to direct and even reflected beam should be avoided without eye protection with class 3 or 4 lasers. Injuries to the eye can occur within moments with exposure to class 3 lasers emitting powers greater than 5 mW, and protective mechanisms of blinking or looking away are ineffective with such high powers. Labeling of the devices may not be accurate. The laser institute of America also cautions against pointing a laser at a mirror-like surface as the reflected beam may act like a direct beam on the eye [3,4].

**Figure 2:**
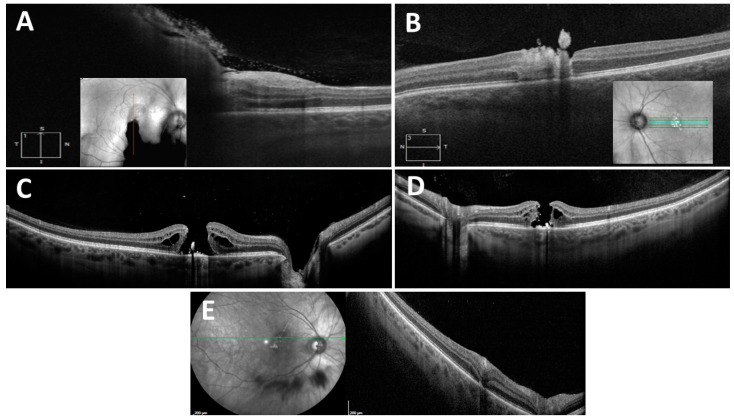
Optical coherence tomography (OCT) images taken at presentation show preretinal hemorrhage in the right eye (**A**) and retinal disruption with preretinal hyper-reflective lesion in the left eye (**B**). One month later, the OCT images demonstrate the development of full-thickness macular holes in the right (**C**) and left eye (**D**), and retinal pigment epithelium clumping and outer retinal atrophy in curvilinear streak areas in the right eye (**E**). While laser-induced macular holes may spontaneously close, surgical intervention may be necessary for persistent cases and may result in increased visual acuity [4]. Spectral domain OCT is reportedly a sensitive tool for evaluating retinal damage and the subsequent evolution of retinal changes [5].
